# Experimental Study of Flexural Behavior of Reinforced Concrete Beam Strengthened with Prestressed Textile-Reinforced Mortar

**DOI:** 10.3390/ma13051137

**Published:** 2020-03-04

**Authors:** Jongho Park, Sun-Kyu Park, Sungnam Hong

**Affiliations:** 1 Department of Civil, Architectural and Environmental System Engineering, Sungkyunkwan University, Suwon 16419, Gyeonggi-do, Korea; rhapsode@skku.edu; 2 Department of Ocean Civil Engineering, Gyeongsang National University, Tongyeong 53064, Gyeongsangnam-do, Korea

**Keywords:** textile reinforced mortar, prestressed textile, failure mode, flexural behavior, flexural stiffness, strengthening efficiency, AR-glass textile, carbon textile

## Abstract

In this study, nine specimens were experimentally tested to analyze the strengthening efficiency of textile-reinforced mortar (TRM) and the difference in flexural behavior between prestressed and non-prestressed TRM-strengthened reinforced concrete beam. The test results show that TRM strengthening improves the flexural strength of TRM-strengthened reinforced concrete beams with alkali-resistant-(AR-) glass textile as well as that with carbon textile. However, in the case of textile prestressing, the strengthening efficiency for flexural strength of the AR-glass textile was higher than that of the carbon textile. The flexural stiffness of AR-glass textiles increased when prestressing was introduced and the use of carbon textiles can be advantageous to reduce the decreasing ratio of flexural stiffness as the load increased. In the failure mode, textile prestressing prevents the damage of textiles effectively owing to the crack and induces the debonding of the TRM.

## 1. Introduction

Severe problems concerning the safety of reinforced concrete (RC) structures arise owing to durability-degrading factors, such as cracking and scaling/spalling of concrete and corrosion of steel reinforcements. These factors occur as a result of various changes in the external environment, such as the increased service life and climate change. Substantial financial resources are required in repairing and strengthening the performance and durability of structures [1], and several researchers have investigated various repair/strengthening methods [2,3,4,5,6]. The textile-reinforced mortar (TRM) method is a structural strengthening method that involves producing textiles with an excellent tensile strength and chemical resistance, such as carbon, alkali-resistant- (AR-) glass, aramid, and basalt fiber, and attaching these materials onto the surface of the concrete structure using mortar, an inorganic material [7]. Unlike fiber-reinforced polymers, which reinforce fibers using polymer materials, textiles are composed of fibers, therefore, they have free formability and are applicable to various structures, including arches, columns, beam-column joints, and masonry structures [8].

Several researchers have attempted to understand the behavior of TRM. Uniaxial tensile tests were conducted to understand the behavior of TRM composites. The detailed behavior of TRM was identified [9,10] and a modeling technique was proposed [11]. The bond behavior between TRM and the concrete substrate is also important in determining the performance of TRM-strengthened RC beam. A bond model of the load carrying capacity was derived for pure shear loading [12], experimentally study for the bond was conducted and effective anchorage length was derived for fibers/matrix interface [13], and the bond-slip relation was predicted considering the loss of bond between PBO-FRCM system and the concrete substrate [14]. The flexural behavior of the structure which was strengthened with TRM was also investigated [15,16,17,18,19,20,21,22,23,24]. Herbrand et al. [25] applied a carbon textile-reinforced mortar to real bridge. The experimental results indicated increasing the fatigue and ultimate strength of bridge. Koutas et al. [7] reported the state-of-the-art review on the strengthening of concrete structure with TRM including studies for flexural, shear, confinement, and seismic retrofitting. 

The designing and strengthening of TRM, however, is still difficult because such an uncertainty involves errors that may occur during weaving, material application, and the construction process. Theoretical studies were conducted to predict the flexural strength of TRM-strengthened RC beam [17,18,20,24]. An efficient factor is applied in the designing of strengthening TRM to consider the poor bond between textile and mortar [26,27]. ACI Committee 549 [28] proposes an effective tensile strain to limit the ultimate tensile strain of fabric-reinforced cementitious matrix (FRCM). Flexural strength was predicted according to ACI 549.4R and the results underestimated the experimental results [19,29]. 

A method of manually stretching the textile or fixing both sides of the textile was adopted during the construction stage to reduce this uncertainty regarding TRM strengthening [15,27]. The method of stretching and fixing the textiles has the advantages of preventing the local bending of the textiles, which can occur when the textiles are placed on the mortar, and accurately maintaining the reinforced axis [27,30]. This method, however, has a disadvantage: the axial load applied to the textile is not constant. Consequently, some researchers analyzed the performance of a structure by introducing a prestress to the textile [31,32,33,34,35]. Reinhardt et al. [31] performed a four-point flexural and pull-out test to investigate the effects of prestressed AR-glass and carbon textiles. The impregnated carbon textile was more suitable for prestressing than the plain carbon textile, and the glass textile was adequate for prestressing in cases of both plain and impregnated textiles. In the prestressed textile, the modulus of rupture increased and the deflection and crack width after the initial crack occurs could be minimized. Meyer and Vilkner [32] introduced prestressing in an aramid fiber mesh and conducted a three-point flexural test, and the flexural behavior was analyzed according to the presence or absence of epoxy end blocks at both ends of the mesh. In the absence of epoxy end blocks, the mesh behaved as unbonded tendons, and in the presence of the end blocks, a substantial increase in the flexural capacity and crack control owing to prestressing was observed. Zhou et al. [34] and Du et al. [33] carried out uniaxial tensile tests on textile composite specimens with a prestressed textile, and the test results indicated that the first-crack stress and tensile strength increased. Du et al. [35] introduced a prestress on a textile and conducted a four-point flexural test, and the experimental results demonstrated that the first-crack and ultimate stresses increased; however, the effect was more substantial in terms of increasing the first-crack stress.

The studies of prestressed textile reviewed above were conducted for textile-reinforced concrete, which differs from TRM-strengthened reinforced concrete beams in terms of structural behavior. Therefore, in this study, a prestress was introduced to a textile of the TRM-strengthened beam. When prestressing was introduced into the TRM, it is expected that not only can the textile be placed exactly at the designed position, but also the limit of the effective strain or efficient factor of the textile can be raised. The flexural behaviors and material characteristics were analyzed for the prestressed and non-prestressed specimens. This result is expected to provide structural stability and a high strengthening efficiency for TRM-strengthened RC beams.

## 2. Experimental Program

### 2.1. Materials

**Figure 1:**
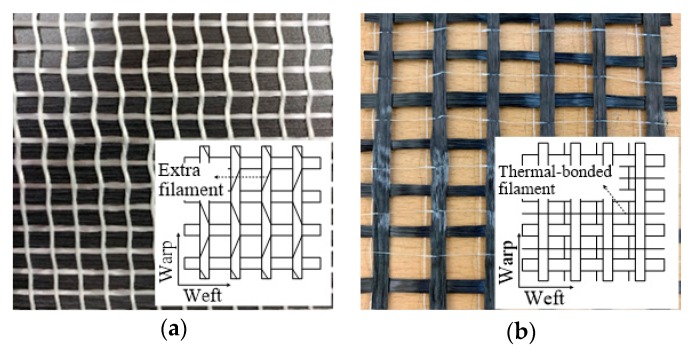
Textiles: (**a**) AR-glass textile; (**b**) carbon textile.

Two types of textiles were used: AR-glass and carbon textiles. AR-glass textile was improved the durability in the alkalinity of cement materials because they contain 16.5% zircon [36,37]. The AR-glass and carbon textiles have 8 mm × 8 mm and 10 mm × 10 mm intervals, respectively. Figure 1 shows the textiles that were used in this study. In the AR-glass textile, warp and weft roving were stacked up and down, and extra filaments were crossed along the warp roving to hold the roving firmly. In the carbon textile, the weft roving overlapped the warp roving, and this roving was fixed by thermal-bonded filaments, which maintained only the shape. The properties of each textile are shown in Table 1. Textiles were cut smaller than the width of the specimen so that AR-glass textile had twelve rovings, and Carbon textile has six rovings per one textile.

Ready-mixed concrete with a specified concrete strength of up to 35 MPa was used for the RC matrix. For the TRM-strengthened part, polymer mortar with a specified concrete strength of up to 45 MPa was used. Table 2 and Table 3 list the mix proportions of concrete and polymer mortar, respectively, and Table 4 shows the detailed specifications of the polymer mortar [38].

### 2.2. Specimens and Test Set-Up

**Figure 2:**
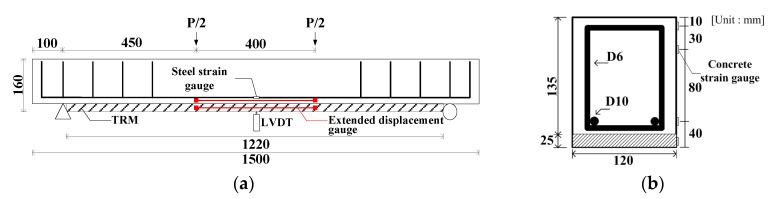
Specifications of the study specimens used in the study. (**a**) Frond view; (**b**) cross-section view at center.

**Figure 3:**
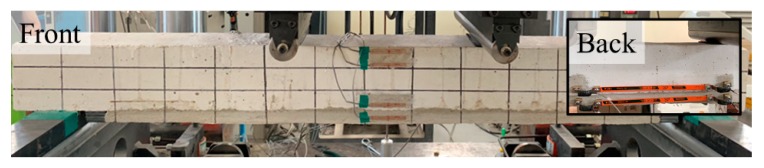
Four-point loading test setup.

An RC beam and eight TRM-strengthened beam specimens were fabricated. Figure 2 shows the detailed specifications of the specimens. Each specimen had a length of 1500 mm and a width of 120 mm. The length of TRM strengthening was 1220 mm considering the support point and length of the zig. The RC beam specimen had a height of 135 mm whereas the TRM-strengthened beam specimens had a height of 160 mm, including TRM part, which was 25 mm in height. The thickness of the TRM part was decided to provide sufficient mortar thickness between the textile strengthening layer. The RC beam specimen was fabricated with a bottom cover thickness of 5 mm, assuming that cross-sectional damage occurred. Steel bars with diameters of 9.53 mm and 6.35 mm for tensile reinforcement and stirrup, respectively, were used. Stirrups were placed at 80 and 100 mm intervals, excluding the pure bending section. All the steel reinforcements had a tensile strength of 400 MPa and a modulus of elasticity of 200 GPa. The span of the specimen was 1300 mm, and the pure bending section was 400 mm in the middle. A four-point loading test with a displacement control of 0.1 mm/s was performed using a 2000 kN of universal testing machine. A linear variable differential transformer (LVDT), three concrete strain gauges, and two steel strain gauges were installed at the center. An extended displacement gauges, which was composed of one thin steel plate with a length of 30 0mm and two Pi-shaped displacement transducer with a length of 50 mm and capacity of 5 mm, were attached to the TRM part and RC beam just above the strengthening part. The extended displacement gauge measures the deformation of TRM. Figure 3 shows the four-point loading test setup.

The textile reinforcement ratio ($\rho_{f})$ of the specimens was compared with the balanced textile reinforcement ratio ($\rho_{fb})$, which was calculated [39,40] by limiting the effective tensile strain of each textile to 0.012 [28]. Analysis of balanced textile reinforcement ratio was performed based on the following assumptions: (1) plane sections remain plane after loading; (2) The bond between concrete, mortar, steel reinforcement and textile is perfect; (3) the ultimate compressive strain of concrete is 0.003. The neutral axis depth at the time of the balance failure of the TRM strengthened RC beams can be expressed as shown in Equation (1):

(1) $$c_{b}=\frac{\varepsilon_{cu}}{\varepsilon_{cu}+\varepsilon_{fe}}d_{f}$$

where $c_{b}$ is the neutral axis depth at balanced failure, $\varepsilon_{cu}$ is the ultimate compressive strain of the concrete, $\varepsilon_{fe}$ is the effective tensile strain (=0.0012) and $d_{f}$ is the effective depth of textile.

Then, balanced textile reinforcement ratio and balanced area of textile reinforcement can be expressed as follows:

(2) $$\rho_{fb}=\frac{A_{fb}}{bd_{f}}$$

(3) $$A_{fb}=\frac{\alpha_{1}f_{ck}b\beta_{1}c_{b}-A_{s}f_{y}}{E_{f}\varepsilon_{fe}}$$

where $A_{fb}$ is the balanced area of textile reinforcement, $A_{s}$ is the area of steel reinforcement, $f_{ck}$ is the specified concrete strength, $f_{y}$ is the yield strength of the steel reinforcement, $E_{f}$ is the modulus of elasticity of filament, and $\alpha_{1}$ and $\beta_{1}$ are the concrete stress block factors which was calculated by Equations (4) and (5).

(4) $$\alpha_{1}\beta_{1}=\frac{\varepsilon_{c}}{\varepsilon_{o}}-\left(\frac{1}{3}\right){\left(\frac{\varepsilon_{c}}{\varepsilon_{o}}\right)}^{2}$$

(5) $$\beta_{1}=\frac{4\varepsilon_{o}-\varepsilon_{c}}{6\varepsilon_{o}-2\varepsilon_{c}}$$

where $\varepsilon_{c}$ is the compressive strain in the concrete, $\varepsilon_{o}$ is the compressive strain of unconfinement concrete ($1.7f_{ck}/E_{c}$), $E_{c}$ is the modulus of elasticity of concrete ($8,500\sqrt[{3}]{f_{ck}+4}$).

Approximately 20% of the textile reinforcement ratio compared with the balanced textile reinforcement ratio was named “Lo1,” 60% was named “Lo2,” and 130% was named “O.” Table 5 lists detailed information regarding the specimens. In Table 5, *Lo* represents the low textile reinforced, *O* represents the over textile reinforced, and *P* represents the prestressed specimen. 

The strengthening process of the TRM specimen was as follows [38]: (1) the bottom surface of the RC beam was ground with a grid of grooves with of 2-3 mm depth; (2) the RC beam was set up on a strengthening device; (3) a primer was applied; (4) 6-mm-thick polymer mortar was poured using a trowel; (5) the textile was placed and pressed into the mortar for the non-prestressed specimens, whereas the textile was fixed on the clamp device at each side for the prestressed specimen; (6) the textile was prestressed parallel to the reinforcing axis to 5% of the tensile strength for the prestressed specimens; (7) a second layer of 6 mm thick polymer mortar was poured, and hand pressure was applied to cause the mortar to penetrate the textile; (8) steps (5)–(7) were repeated until all the designed textile layers were placed; (9) 6-mm-thick mortar was poured after the last textile was placed; (10) the prestressed TRM-strengthened beam specimen was cured for one day with the textiles fixed by using anchors and clamps. 

**Figure 4:**
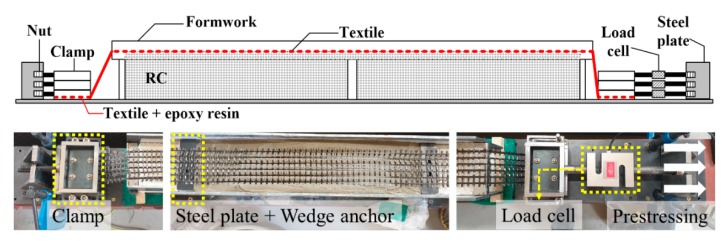
Prestressed textile.

The prestressing system of this study was shown in Figure 4. Before prestressing, both ends of the textile were impregnated with epoxy resin so that the prestress load can be evenly distributed over the roving through the clamp. The textile was bent as it passed through both ends of the formwork and front of the clamp, respectively. This is a method for prestressing in a limited space by changing the direction of the textile and can also introduce a prestressing force on the textile regardless of the workspace which is limited in the real structure. The prestressing was introduced by tightening the nuts at both ends after the textile was fixed to the clamp. Prestressing force was measured by the load cell. After the designed prestressing force was introduced into all textiles, the steel plate and wedge anchors were used to fixing the textiles.

## 3. Experiment Results and Discussion

**Figure 5:**
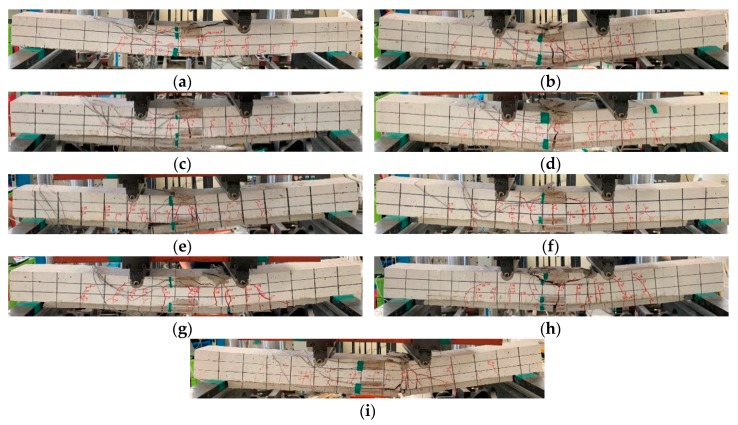
Crack and failure mode: (**a**) RC; (**b**) ARLo1; (**c**) ARLo1P; (**d**) ARLo2; (**e**) CaLo1; (**f**) CaLo1P; (**g**) CaLo2; (**h**) CaLo2P; (**i**) CaO.

**Figure 6:**
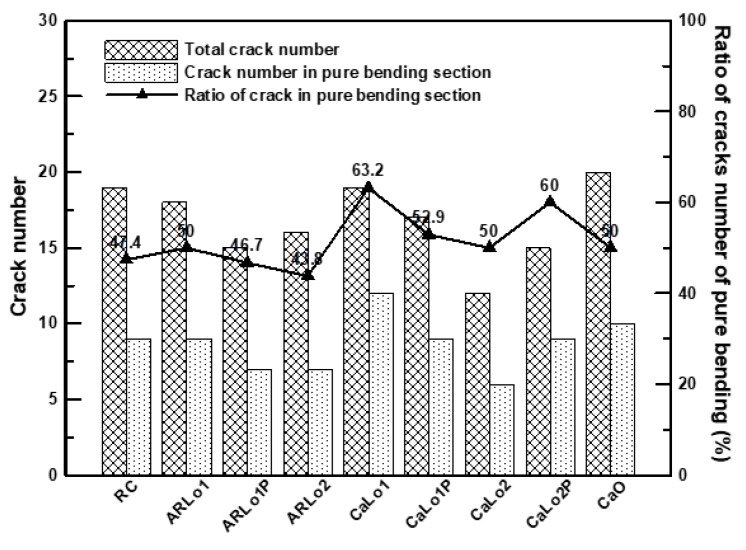
Crack numbers of all the specimens in the RC section.

**Figure 7:**
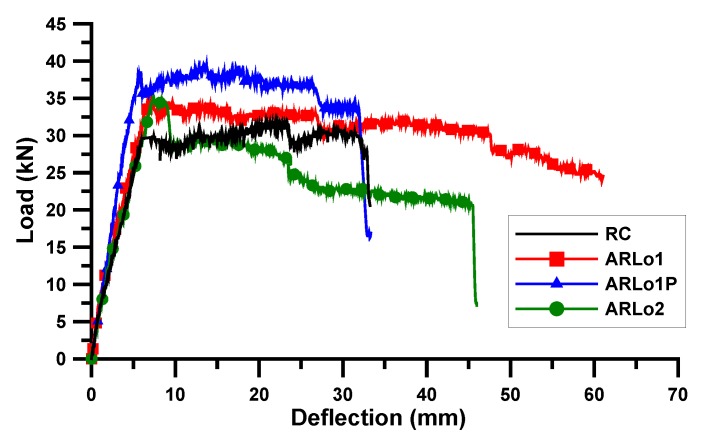
Load and deflection curve: AR-glass-TRM specimen.

**Figure 8:**
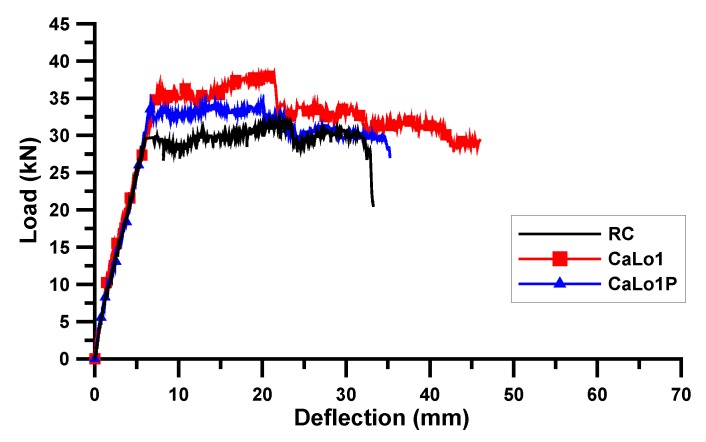
Load and deflection curve: Lo1 series of carbon-TRM specimen.

**Figure 9:**
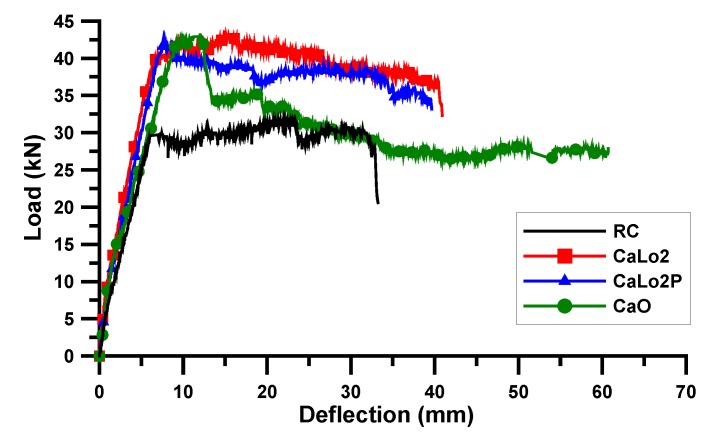
Load and deflection curve: Lo2 and O series of carbon-TRM specimen.

A static loading test was conducted for the AR-glass- and carbon-TRM-strengthened beam specimens. Table 6 shows the test results of the cracking load ($P_{c}$) and deflection ($\delta_{c}$), yield load ($P_{y}$) and deflection ($\delta_{y}$), ultimate load ($P_{u}$) and deflection ($\delta_{u}$), and failure mode. Except for ARLo1, the crack loads of the TRM-strengthened beam specimens were lower than those of the RC beam specimen because the surface of RC beam of TRM specimen was damaged by surface grinding. The cracks and failure were observed through visual inspection and are shown in Figure 5 and Figure 6. The load and deflection curves are shown in Figure 7, Figure 8 and Figure 9.

### 3.1. Crack and Failure

Figure 5 shows a specimen at failure. Each specimen showed a flexural crack pattern similar to that of the conventional RC beam.

Figure 6 displays the total number of cracks in the RC beam specimens and the number of cracks in the pure bending section. The number of cracks was measured after specimen failure and the end of loading. In all specimens, except ARLo1P and ARLo2, more than 50% of the cracks occurred in the pure bending section. This result indicates that TRM strengthening is advantageous for the uniform distribution of cracks [7,35,38,41]. In the ARLo2 specimen, the ratio of the cracks in pure bending decreased. This trend occurred because several textiles (3 lamination × 3 layers) were reinforced in a small space, which reduced the textile-mortar bond areas; therefore, the textiles could not resist the load adequately.

The carbon-TRM-strengthened beam specimens had cracks that were more concentrated at the center compared to those of the AR-glass-TRM-strengthened specimens because the carbon fibers generally had a higher tensile strength and modulus of elasticity. In CaLo1P and CaLo2, a trend of fewer cracks in pure bending was observed. However, in the CaLo2P specimen, the crack distribution in the pure bending section was uniformed. This behavior occurred because the prestressing force increased to three times more than that of CaLo1P so that the efficiency of TRM strengthening increased. The specific reasons are explained in Section 3.2.2.

In all the TRM-strengthened beam specimens, only flexural cracks occurred until the yield load was reached. After the yield load was attained, the debonding of the TRM part and the rupture of the textile occurred, and the specimen failed via the crushing of concrete. For the specimens with no textile rupture, it was assumed that the slippage or deformation of the textile occurred continuously. Table 6 presents the failure modes of all the specimens in the order of their observation. *C*, *R*, and *D* denote concrete crushing, textile rupture, and debonding of the TRM part, respectively. In all the specimens except ARLo1, ARLo2, and CaLo2, the debonding of the TRM part occurred first. The main failure mode of the ARLo1 and ARLo2 specimens were textile rupture. CaLo1 was the only carbon-TRM-strengthened specimen that experienced textile rupture. Textiles are vulnerable to damage owing to exposure to the outside surface where cracks occur [11]. In other words, CaLo1 was vulnerable to damage because its textile reinforcement ratio was minimum compared to those of other carbon-TRM-strengthened specimens. AR-glass textiles appear to be more vulnerable to damage. However, no rupture occurred, in the prestressed specimen of ARLo1P and CaLo1P. In textiles of CaLo2, no damage was observed, and concrete crushing occurred as the crack width increased.

### 3.2. Load and Deflection Relationship

#### 3.2.1. AR-Glass Textile

Figure 7 displays the load and deflection curves for the AR-glass-TRM-strengthened beam specimens. The yield and ultimate loads of the RC beam specimen were 29.59 and 31.81 kN, respectively. The yield load of ARLo1 was 35.51 kN, which was 20.0% higher than that of the RC beam specimen (the reference specimen). The yield and ultimate loads of ARLo1P were 38.46 and 39.45 kN, respectively, which were 30.0% and 24.0% higher than those of the RC beam specimen, and the yield load of ARLo1P was 8.3% higher than that of ARLo1. The yield load of ARLo2 was 35.26 kN, which was 19.2% higher than that of the RC beam specimen but had a lower yield load than those of ARLo1 and ARLo1P. The strengthening efficiency of the textile was higher when more textiles were laminated in one layer, as exhibited by ARLo1, but the strengthening efficiency decreased owing to the low bonding efficiency between the textile and mortar when multiple textiles (3 lamination × 3 layers) were constructed in a small space, as exhibited by ARLo2 [34,38].

The prestressed ARLo1P specimen, in which a prestress of 5% of tensile strength was introduced, demonstrated a higher strengthening efficiency than the non-prestressed ARLo1 specimen. Thus, textile prestressing significantly contributed to the increase in the strength of the TRM-strengthened beam. In particular, the failure modes presented in Table 6 indicate that textile rupture occurred in the non-prestressed specimens, whereas the debonding of the TRM part occurred in the prestressed specimen. In other words, textile prestressing prevents textile damage owing to cracks and allows textiles to exhibit sufficient strength by inducing debonding of the TRM reinforcement.

#### 3.2.2. Carbon Textile

Figure 8 and Figure 9 show the load and deflection curves of the carbon-TRM-strengthened beam specimens. The yield and maximum loads of CaLo1 were 36.49 and 38.22 kN, respectively, which were 23.3% and 20.2% higher than those of the RC beam specimen. The yield load of CaLo1P was 34.4 kN, which is 16.3% higher than that of the RC and 5.7% less than that of the CaLo1 specimens. The Lo1 series of the carbon-TRM-strengthened beam specimens did not show any increase in strength after prestressing. In contrast, the yield load of CaLo2P was 42.66 kN, which is 44.2% and 5.5% higher than those of the RC and CaLo2 specimens, respectively. The yield and maximum loads of CaLo2 were 40.44 and 43.04 kN, respectively, which were 36.7% and 35.3% higher than those of the RC beam specimen. The yield and maximum loads of CaO were 41.92 and 43.15 kN, respectively, which is 41.7% and 35.6% higher than those of the RC beam specimen, but they are similar to those of CaLo2 and CaLo2P, indicating a low strengthening efficiency. 

The strengthening efficiency of CaLo2P increased owing to textile prestressing, CaLo1P did not exhibit an increase in strength. In other words, the Lo1 series of the carbon-TRM-strengthened beam specimens had a different tendency compared with the Lo1 series of the AR-glass-TRM-strengthened beam specimens with the similar textile reinforcement ratio. This difference existed because the bond condition between the carbon roving and mortar was weaker than that between the AR-glass roving and mortar; in particular, the bond condition of the plain carbon roving was further lowered under prestress [31]. Additionally, the difference between the textile configurations, as shown in Figure 1, should be considered. The carbon textile form was easily distorted when the textile received a prestressing force, and the possibility of distributing of the load uniformly on the textiles became low; hence, the possibility of damaging the carbon fibers was high. Therefore, the CaLo2P specimen demonstrated a high strength owing to sufficient textile reinforcement ratio to compensate for the low-bond performance in prestressing but exhibited a lower strengthening efficiency compared to that of the AR-glass textile.

### 3.3. Flexural Stiffness

**Figure 10:**
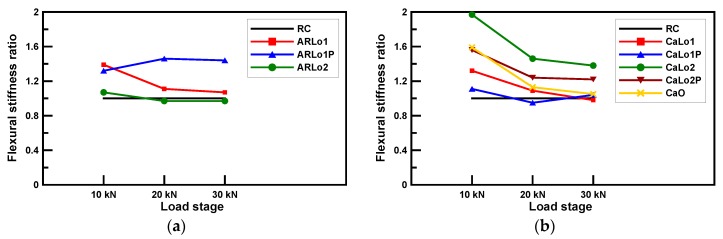
Comparison of the flexural stiffness ratio of all the specimens: (**a**) AR-glass textiles; (**b**) carbon textiles.

Figure 10 shows the comparison of flexural stiffness ratio which was the value obtained by dividing the flexural stiffness of each specimen by that of RC beams. Flexural stiffness is a significant factor in determining the deflection characteristics of RC. The deflection can be calculated by substituting the effective moment of inertia into the elastic deflection formula of the simple beam under the three assumptions in Section 2.2 [42]. Therefore, the effective moment of inertia can be identified by using the load and deflection in the test results. Equation (6) shows the center deflection of a four-point-loading simple beam, and Equation (7) expresses the effective moment of inertia of the section determined by Equation (6).

(6) $$\delta_{c}=\frac{Pa}{48E_{c}I_{e,a}}\left(3l^{2}-4a^{2}\right)$$

(7) $$I_{e,P}=\frac{Pa}{48E_{c}\delta_{c}}\left(3l^{2}-4a^{2}\right)$$

where $\delta_{c}$ is the deflection at the center, P is the load, a is the length from the support to the loading point, l is the length of span, $E_{c}$ is the elastic modulus of concrete, and $I_{e,P}$ is the effective moment of inertia when a load (P) is applied.

In the AR-glass-TRM-strengthened beam specimens, the flexural stiffness of ARLo2 was lower than that of the RC beam specimen, except at 10 kN loading. The deformation of AR-glass textile occurred as slippage because the textile-mortar bond was insufficient; thus, the AR-glass textile in ARLo2 had no significant effect on the flexural stiffness. The flexural stiffness of ARLo1P was less than that of ARLo1 at 10 kN loading, but at a higher loading, the flexural stiffness of ARLo1P was more than 30% of that of ARLo1, demonstrating that the prestressing effect increases the flexural stiffness. Among carbon-TRM-strengthened beam specimens, CaLo1 and CaLo1P had no significant effect on the increase in flexural stiffness even though both specimens showed yield loads 19.8% higher than that of the RC beam specimen on average. This insignificant effect was observed because the cross-sectional area of the reinforced textile was minimal compared to that of the RC and mortar; hence, the effect of low textile reinforcement ratio on the increase in flexural stiffness was small [41], and the prestressing performance was poor in plain textile [31]. CaLo2P and CaO had similar flexural stiffness values at 10 kN loading, but as the load increased, the flexural stiffness of CaLo2P became approximately 10% higher, and CaO had a flexural stiffness similar to that of the RC beam specimen at 30 kN loading. CaLo2 showed a 15.4% higher flexural stiffness than CaLo2P on average, but this difference decreased continuously as the load increased. The flexural stiffness of CaLo2 was decreased by 25.9% and 5.5% for each load step, whereas CaLo2P was decreased by 20.5% and 1.6%. In the case of CaO, the flexural stiffness was decreased by 28.9% and 7.1% for each load steps. Therefore, carbon textile prestressing can be more advantageous to reduce the decreasing ratio of flexural stiffness as the load increased.

### 3.4. Strain

**Figure 11:**
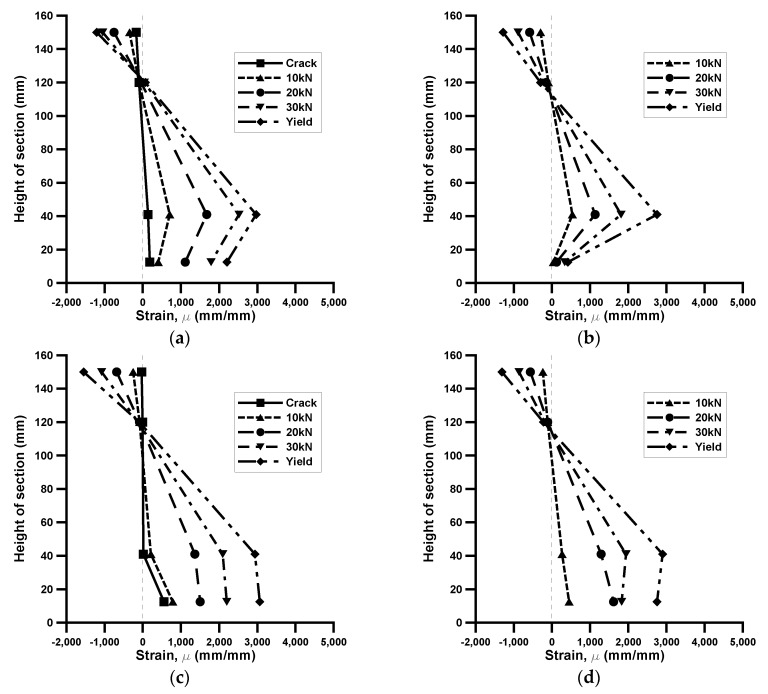
Strain distribution of different load levels: (**a**) ARLo1; (**b**) ARLo1P; (**c**) CaLo2; (**d**) CaLo2P.

The strains of the prestressed and non-prestressed TRM-strengthened beam specimens according to the cross-sectional height are shown in Figure 10. The strains at 150 and 120 mm were measured using concrete strain gauges. The strains at 40 mm were measured using steel strain gauge and at bottom points was measured using the extended displacement gauge. The Figure 11a,b displays the changes in strain in ARLo1 and ARLo1P, respectively. The strain variations at the top of the concrete were similar in all the load steps. In ARLo1P, the strain of steel reinforcements and TRM was smaller than that in ARLo1. Particularly, the strain in the TRM part of ARLo1P exhibited no significant change. Similar trends can be observed for CaLo2 and CaLo2P in Figure 11c,d, respectively. The steel reinforcement strain in CaLo2 from the formation of the crack to 10 kN was less than that of the CaLo2P, but the steel reinforcement strain in CaLo2P became less than that of CaLo2 as the load increased. The strain in the TRM part of CaLo2 was similar compared with that of CaLo2P until the loading reached 20 kN; however, the strain in CaLo2P subsequently became smaller than that of CaLo2. Therefore, introducing textile prestressing is expected to enhance the uniformity in the load distribution of the textiles and steel reinforcement, and to increase the strengthening efficiency of the structure.

## 4. Conclusions

1. The failure modes of TRM-strengthening beams can be classified into the debonding of the TRM part and the rupture of the textile. In this study, textile rupture occurred in the non-prestressed TRM-strengthened beam specimens that contained AR-glass and carbon textiles at low reinforcement ratios. However, in the comparison group where prestressing was introduced textile rupture did not occur and debonding occurred first. Therefore, the textiles exhibited a high performance as the debonding of the TRM part was induced with the introduction of prestressing. In addition, TRM strengthening has advantages for the uniform distribution of cracks.
2. The prestressed AR-glass-TRM-strengthened specimen (ARLo1P) had a yield load that was 8.3% higher than that of the non-prestressed specimen (ARLo1). The carbon-TRM-strengthened beam specimens did not demonstrate any prestressing effect at low textile reinforcement ratio (CaLo1 and CaLo1P), but when the textile reinforcement ratio was sufficient (CaLo2 and CaLo2P), the prestressed TRM-strengthened beam specimen exhibited a 5.5% higher yield load. Hence, textile prestressing improved the flexural performance effectively, but the strengthening efficiency of AR-glass textile was higher than that of the carbon textile.
3. For the AR-glass-TRM-strengthened beam specimens, the prestressed specimens comparatively exhibited a higher flexural stiffness as the load increased. In prestressed carbon-TRM-strengthened beam specimen, the decrease in flexural stiffness was smaller than that in the non-prestressed specimens. Thus, the flexural stiffness of the AR-glass textile increased when prestressing was introduced, and the use of the carbon textile enhance the stability of structures.

In this study, however, differences in flexural behavior were observed owing to the differences in the shapes of glass and carbon textiles. Therefore, the appropriate selection of textiles for structural reinforcement is crucial, and further research is required on the standardization of textiles to generalize experimental results.

## Figures and Tables

**Table 1:** Detailed specifications of the AR-glass and carbon textiles.

Properties and Geometric Parameters	AR-Glass Textile	Carbon Textile
Tensile strength of filament (MPa)	1789	4900
Modulus of elasticity of filament (GPa)	68	230
Elongation of filament	0.0262	0.022
Number of filaments per roving	1,600	12,000
Diameter of filament (µm)	14	7
Area per one textile (mm^2^)	2.952	2.772

**Table 2:** Mix proportion of concrete.

W/B (%)	Unit Weight (kg/m^3^)						
	Cement	Water	Fine Aggregate	Coarse Aggregate	Fly Ash	Blast Furnace Slag	Water Reducer
35.8	319	163	780	898	68	68	4.1

**Table 3:** Mix proportion of polymer mortar.

W/M ^1^ (%)	Content Per 1 Bag of 25 kg (%)					
	Cement	Fine Aggregate ^2^	PVA Fiber ^3^	Acrylate Copolymer	EA ^4^	Water Reducer
19	<50	35~40	>1	>3	>5	<1

^1^ Water-mortar ratio; ^2^ Silica sand; ^3^ Polyvinyl alcohol fiber; ^4^ Hexacalcium hexaoxotris expansive admixture.

**Table 4:** Detailed specifications of the polymer mortar.

Strength Type	Experimental Value (MPa)	Standard of KS (MPa)
Flexural	8	more than 6
Bond ^1^	1.5	more than 1

^1^ The bond strength was increased by 20% with primer.

**Table 5:** Detailed specification of study specimens.

Name	Strengthening Material	Textile		$\rho_{f}$ **/** $\rho_{fb}$	Prestress Load
		Lamination ^1^	Layer ^2^		
RC	**-**	**-**	-	-	-
ARLo1	AR-glass fiber	3	1	20.5%	-
ARLo1P		3	1		792N
ARLo2		3	3	61.48%	-
CaLo1	Carbon	1	1	21.59%	-
CaLo1P		1	1		679N
CaLo2		3	1	64.85%	-
CaLo2P		3	1		2037N
CaO		2	3	129.62%	-

^1^ Lamination is the stacking of textiles per one layer; ^2^ Layer is a placing of laminated textile at regular intervals.

**Table 6:** Test results.

Name	Experimental Results						Failure Mode ^1^
	$P_{c}\;(\mathrm{kN})$	$\delta_{c}\;(\mathrm{mm})$	$P_{y}\;(\mathrm{kN})$	$\delta_{y}\;(\mathrm{mm})$	$P_{u}\;(\mathrm{kN})$	$\delta_{u}\;(\mathrm{mm})$	
RC	5.68	0.75	29.59	6.02	31.81	21.94	C
ARLo1	6.91	0.77	35.51	6.96	-	-	R + D, C
ARLo1P	-	-	38.46	5.9	39.45	13.36	D, C
ARLo2	1.72	0.29	35.26	7.36	-	-	R, C
CaLo1	3.82	0.55	36.49	8.05	38.22	20.78	D, R, C
CaLo1P	4.19	0.46	34.4	6.71	-	-	D, C
CaLo2	2.47	0.09	40.44	6.97	43.04	15.11	C
CaLo2P	-	-	42.66	7.76	-	-	D, C
CaO	-	-	41.92	9.14	43.15	12	D, C

^1^*C*, *R*, and *D* denote concrete crushing, textile rupture, and debonding of the TRM part, respectively.
